# Low intensity focused ultrasound modulates monkey visuomotor behavior

**DOI:** 10.1186/2050-5736-3-S1-O25

**Published:** 2015-06-30

**Authors:** Thomas Deffieux, Nicolas Wattiez, Mickael Tanter, Pierre Pouget, Jean-Francois Aubry, Youliana Younan

**Affiliations:** 1 Institut Langevin, Paris, France; 2 Institute of Brain and Spinal Cord, Paris, France

## Background/introduction

*In vivo* feasibility of using low intensity focused ultrasound to transiently modulate the function of regional brain tissue has been recently tested in anesthetized rabbits and rodents. In this work, antisaccade latencies have been modulated with non-invasive low intensity focused ultrasound (FUS) in the brain of two awake Maccaca Mulatta monkeys (Y and L).

## Methods

Animals were specifically trained in an antisaccade (AS) paradigm: after fixation of a central visual stimulus on a screen, this stimulus disappeared and a peripheral target appeared, right or left. Monkeys were trained not to look at this peripheral target but instead initiate a saccade towards the opposite direction. Eye movements were recorded with an infra-red eye tracker (Eyelink 1k, SR-Research, Ontario, Canada), and eye position was digitized and stored for off-line analyses. In each experiment session, animals performed a total of 3 blocks of AS training per session. First, monkeys performed a 100 trials block of AS (50 for each side) as baseline. Then, a second block of 400 trials was performed: 360 trials without US (180 for each side) and 40 trials with US (20 for each side) were presented. Trials with US were pseudo-randomly interleaved with trials without US. A final block of 100 trials was performed as a post-test. Monkey Y performed 10 sessions and monkey L 12 sessions.

Control sessions, using identical procedures, with the transducer positioned over the pre- motor cortex instead of the left FEF were performed (Monkey Y – 8 sessions, Monkey L – 7 sessions). Finally, sham sessions inspired from TMS sham experiments were performed: the ultrasound transducer was moved 4 cm away from the animal’s head so that ultrasound could not reach previous target (Monkey Y – 5 sessions, Monkey L – 2 sessions). FUS consisted in continuous 100ms sonication with a 320 KHz transducer (H115, Sonic Concept, Bothell, WA, USA) focused at the Frontal Eye Field (identified according to stereotaxic coordinates). The estimated derated pressure in the brain was 0.35 ± 0.05 MPa.

## Results and conclusions

Ipsilateral mean AS latencies with ultrasound stimulation were significantly slowed (monkey Y: p= 0.0018; monkey L: p< 0.001) compared to the non-stimulated condition (monkey Y: noUS= 221 ms US= 235 ms; monkey L: noUS= 239 ms US= 269 ms). For the two animals, contralateral mean AS latencies were not significantly slowed (t-test: monkey Y: p> 0.8; monkey L: p>0.6) compared to the non-stimulated condition. Focused ultrasound stimulation applied within a control pre-motor cortex did not significantly affect ipsilateral anti-saccade latencies (t-test: monkey Y: p> 0.69; monkey L: p> 0.1) or contralateral latencies (monkey Y: p> 0.11; monkey L: p> 0.74). In both monkeys, sham focused ultrasound did not interfere with ipsi- or contralateral saccade latency (p> 0.5).

The study demonstrates the feasibility of using focused ultrasound stimulation to causally modulate behavior in the awake non-human primate brain.

